# Yes-associated protein 1 in cancer: bridging mechanical transduction and epigenetic regulation

**DOI:** 10.1080/15384047.2025.2562726

**Published:** 2025-09-21

**Authors:** Tingting Liu, Shuo Yu, Lu Zhang, Wenwen Ji, Guangdong Wang, Na Wang, Mengcong Li, Tinghua Hu, Zhihong Shi

**Affiliations:** a Department of Respiratory and Critical Care Medicine, The First Affiliated Hospital of Xi'an Jiaotong University, Xi'an, China; b Department of General Surgery, The Second Affiliated Hospital of Xi'an Jiaotong University, Xi'an, China; c Department of Tumor and Immunology in Precision Medical Institute, The Second Affiliated Hospital, Xi'an Jiaotong University, Xi'an, China

**Keywords:** Yes-associated protein 1, extracellular matrix, epigenetic modifications, therapy resistance.

## Abstract

Yes-associated protein 1 (YAP1) and its paralog TAZ serve as central mechanotransductive transcription coactivators that integrate mechanical cues from the extracellular matrix, such as stiffness and fluid shear stress, with epigenetic modifications to drive oncogenic processes. They regulate diverse biological functions, including proliferation, metastasis, immune evasion, autophagy, ferroptosis, and metabolism. This review highlights how YAP1/TAZ signaling is modulated by mechanosensitive pathways (Integrin/FAK, Rho GTPases) and epigenetic mechanisms (m6A methylation, DNA methylation), contributing to therapy resistance and disease progression. Targeting the mechano-epigenetic axis of YAP1/TAZ offers promising therapeutic strategies for cancer treatment.

## Introduction

Cancer continues to be a major threat to human health, with significant impacts on global morbidity and mortality. Despite advancements in treatment options, including surgery, chemotherapy, radiotherapy, and targeted therapies, the 5-y survival rate for many cancer patients remains disappointingly low.^1^ This underscores the critical need to decipher the intricate molecular networks that drive tumor progression and identify novel therapeutic vulnerabilities.

Traditionally, cancer research has focused on genetic mutations and biochemical signaling pathways. However, a burgeoning body of evidence highlights the indispensable role of the tumor microenvironment (TME) in oncogenesis.^2^ Beyond biochemical cues, the physical properties of the TME, particularly the increased extracellular matrix (ECM) stiffness and altered mechanical forces, are now recognized as potent drivers of malignant phenotypes.^3^ These mechanical cues are transduced into biochemical signals, a process known as mechano-transduction, that regulate gene expression, cell proliferation, invasion, and stemness.^4^ Mechanical transduction within cancer cells contributes to the hallmark characteristics of solid tumors. Environmental mechanical cues, such as matrix stiffness, fluid stress, stretch forces, and ultrasonic stress, play a pivotal role in regulating the molecular mechanisms that drive cancer cell behavior.^5^ Cells sense mechanical regulators through a series of proteins, which transduce these signals into downstream molecular pathways. This mechano-signaling cascade thereby orchestrates fundamental cellular behaviors critical for cancer progression.^6-9^ Mechanosensitive proteins, including integrins, focal adhesion kinases (FAKs), and cytoskeletal components—are critical for cancer metastasis and progression. They mediate the transduction of mechanical cues into biochemical signals, which in turn drive the aggressive behaviors of tumor cells.^10-13^ The remodeling of ECM activates downstream signaling pathways through the aforementioned mechanosensitive molecules. Consequently, this aberrant physical microenvironment not only further escalates ECM stiffness but also disrupts immune cell infiltration and remodels the genetic epigenetics, collectively fostering tumor progression.^14^

Furthermore, a deeper understanding of tumor mechanobiology not only provides insights into the physical pathogenesis of cancer but also opens up novel avenues for mechano-based therapeutic interventions. Importantly, emerging evidence suggests that mechanical cues can induce lasting epigenetic changes, thereby mechanically reprogramming cancer cell phenotypes and contributing to therapy resistance. The epigenetic regulation, which modulates gene expression without altering the DNA sequence, is increasingly implicated in cancer development, influencing cell fate decisions, plasticity, and response to therapy.^15^ In recent years, epigenetic findings have provided novel fields and revealed interesting phenomena.^16^ The epigenetic changes in Yes-associated protein 1 (YAP1) potentially influence expression and various biological processes. m6A modification plays important roles in gene expression and disease development.^17^ This crosstalk between mechanical transduction and epigenetic regulation forms a critical mechanistic link in tumor progression, and targeting this axis may offer innovative strategies for cancer treatment.

 At the intersection of these two fundamental processes lies the transcriptional coactivator YAP1 and its paralog TAZ. The Hippo pathway is considered the most important pathway in mechanical transduction. YAP1 expression, a central mechano-mediated transcription coregulator, activates a number of downstream molecules to influence biological behaviors.^18^ Various signaling molecules—including integrins, PIEZO1, and TRPV4—not only act as mechano-sensors but also directly interact with YAP1, thereby facilitating its activation and nuclear translocation in response to mechanical cues. Independently, YAP1 is also subject to extensive epigenetic modifications, including DNA methylation,^19^ histone acetylation, and N6-methyladenosine (m6A) RNA methylation,^20^ which finely tune its expression, stability, and transcriptional activity. While the roles of YAP1 in mechano-transduction and epigenetics have been extensively reviewed in isolation, the crosstalk and synergy between these two regulatory layers have not yet been fully elucidated. How mechanical signals influence the epigenetic landscape of YAP1, and conversely, how epigenetic modifications dictate the mechanical response of cancer cells, represents a fascinating and underexplored frontier in oncology.

Autophagy, apoptosis and other typical biological approaches are associated with YAP1 expression to induce the development of diseases.^21^ Numerous metabolic pathways are intricately associated with YAP1/TAZ activity, through which they critically regulate diverse cell behaviors as well as orchestrate processes essential for tissue regeneration and repair.^22^ Here, we concluded recent data providing distinct thoughts about YAP1/TAZ among cellular metabolism, cancer progression, and drug resistance. When these factors are abnormal, YAP1/TAZ signaling pathway is exceptionally activated in multiple diseases, such as cancer, fibrosis, inflammation, and atheromatous disease.^23^ During the development of above diseases, novel phenotypic patterns may be activated, such as apoptosis, autophagy, ferroptosis, and so on.

**Figure 1.**
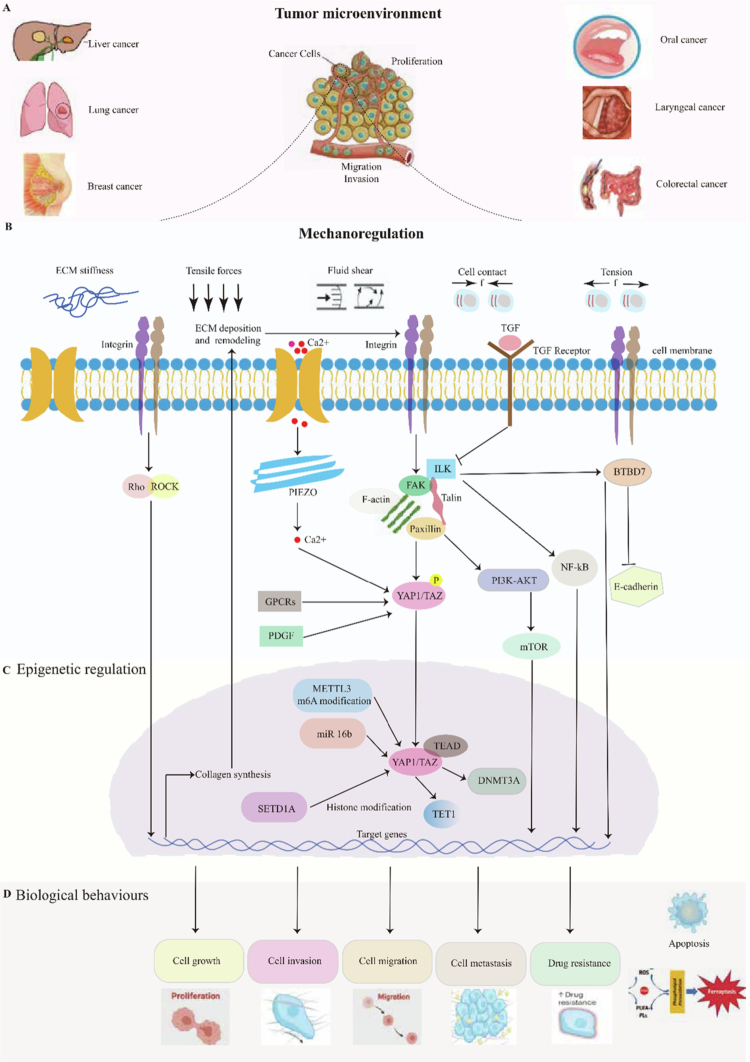
YAP1/TAZ integrates mechanical and epigenetic signals to drive oncogenic programs across multiple cancer types. Schematic overview of the mechano-epigenetic regulatory axis centered on YAP1/TAZ. Mechanical cues such as ECM stiffness, tensile forces, and fluid shear stress are sensed by mechanosensors (integrins, PIEZO channels), which activate downstream signaling pathways (FAK-SRC, Rho-ROCK, and mTOR) that promote YAP1/TAZ nuclear translocation. Nuclear YAP1/TAZ forms a complex with TEAD transcription factors to regulate target genes involved in proliferation, invasion, migration, metastasis, and drug resistance. Concurrently, YAP1/TAZ activity is modulated by epigenetic mechanisms, including DNA methylation (DNMT3A), histone modification (SETD1A), and RNA m6A methylation (METTL3), creating a bidirectional feedback loop that amplifies oncogenic signaling.

As a comprehensive review, we aim to synthesize and critically evaluate the current understanding of YAP1 in cancer biology, with a specific focus on its dual role as a mediator of mechanical transduction and epigenetic regulation. We will explore the upstream and downstream signaling mechanisms of YAP1, its interaction with the ECM, and its modulation through various epigenetic modifications. Furthermore, we discuss the involvement of YAP1 in emerging biological processes such as autophagy, ferroptosis, metabolism, and immune infiltration. By providing a holistic view of YAP1's multifunctional roles, this review seeks to bridge mechanistic insights with therapeutic potential, offering valuable perspectives for future research and cancer treatment strategies (Figure 1).

## YAP1 in pan-cancer: mechanisms and clinical implications

The oncogenic role of YAP1 extends across diverse cancer types, highlighting its universal significance in malignant progression. Beyond its well-characterized functions in liver cancer and lung cancer, YAP1 dysregulation drives pathogenesis in cancers of the breast, gastrointestinal tract, head and neck, and other organs. For instance, in oral squamous cell carcinoma, YAP1 activation is critically implicated in the malignant transformation of premalignant lesions, such as oral submucous fibrosis. Sharma et al demonstrated that YAP1/TAZ signaling confers stem-like properties in OSF-derived cells, thereby bridging stromal fibrosis to overt carcinogenesis.^24^ Similarly, in breast cancer, YAP1 activation is correlated with increased mammographic density and tissue stiffness, driving tumor initiation and therapy resistance.^25^ In colorectal cancer, YAP1 cooperates with DNMT3A to epigenetically silence tumor suppressors, facilitating metastasis.^26^ This pan-cancer perspective underscores YAP1 as a central node that integrates mechanical and epigenetic cues to orchestrate context-dependent oncogenic programs. To visually encapsulate this complexity, we have redesigned our schematic model to map YAP1-associated mechanisms across major cancer types, emphasizing tissue-specific pathways and therapeutic vulnerabilities.

## In-depth mechanistic elucidation of YAP1 and TAZ

### Central role as transcriptional integrators

YAP1 and TAZ are the primary downstream effectors of the highly conserved Hippo tumor suppressor pathway.^27^ They are not DNA-binding transcription factors themselves but are potent transcriptional coactivators that integrate a vast array of extracellular and intracellular signals, including mechanical cues, cellular energy status, mitogenic signals and cellular adhesion, to dictate fundamental cellular processes such as proliferation, differentiation, survival, and migration.^28^ Their dysregulation is a hallmark of cancer, fibrosis, and other proliferative diseases. This section provides a detailed mechanistic overview of their regulation and function.

### Core cytoplasmic regulation: the Hippo kinase cascade

**Figure 2.**
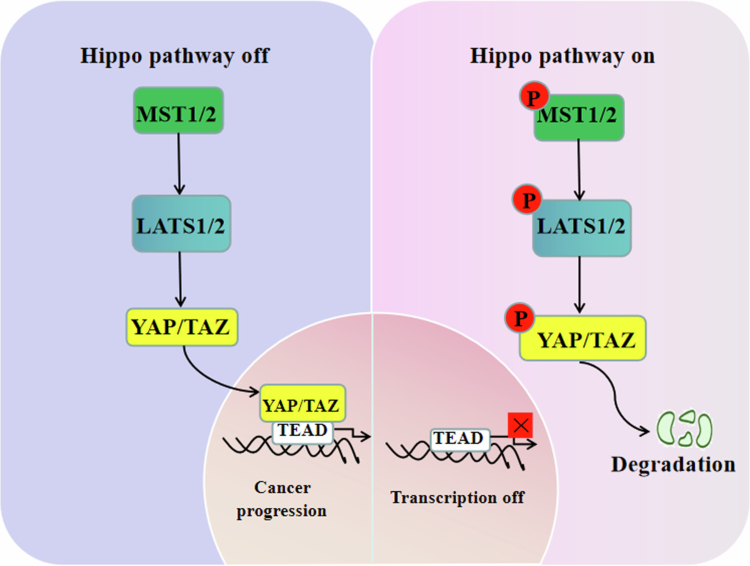
The canonical Hippo pathway regulates YAP/TAZ activity through phosphorylation-dependent nucleocytoplasmic shuttling. When the Hippo pathway is ON (left), the kinase cascade comprising MST1/2 and LATS1/2 is activated. LATS1/2 phosphorylates YAP1/TAZ, leading to their cytoplasmic retention and subsequent degradation, thereby inhibiting their transcriptional activity. When the Hippo pathway is OFF (right), unphosphorylated YAP1/TAZ translocates into the nucleus, where it forms a complex with TEAD family transcription factors. This YAP1/TAZ–TEAD complex activates the expression of protumorigenic target genes that drive cancer hallmarks such as proliferation, survival, and metastasis.

The canonical regulation of YAP1/TAZ is governed by a kinase cascade culminating in their phosphorylation, cytoplasmic sequestration, and degradation. The Hippo pathway is a highly crucial signaling pathway in Drosophila for tumor suppressor factors*.* It is consisted of kinase cascade reactions, including MST1/2 and LATS1/2, in mammals to regulate the phosphorylation of cotranscription factors. YAP1/TAZ is considered the most common cotranscription factor to mediate the malignant biological behaviors of tumor, such as cell proliferation, migration, and invasion.^29^ Previous studies have identified potential mechanisms about the nuclear‒cytoplasmic translocation of YAP1 by controlling its phosphorylation level. When the upstream hippo-pathway kinases MST1/2 and LATS1/2^30^ are phosphorylated, the downstream YAP1 is inactivated and remains in the cytoplasm.^31^ Conversely, once the Hippo pathway is inactivated, YAP1 and TAZ are transferred to the nucleus, promoting the transcription level of downstream genes^32-34^ (Figure 2).

### Non-canonical and Hippo-independent regulation

#### Mechanical stress

During the tumor progression, abnormal mechanical forces, including wall shear stress (WSS), fluid shear stress (FSS) and matrix stiffness, trigger signaling pathways that activate cellular behaviors.^35^ ECM stiffness, which refers to rigidity or elasticity, plays a critical role in regulating essential cellular processes such as diffusion, growth, proliferation, migration, differentiation, and organoid formation. Due to its broad influence, ECM stiffness has been extensively studied in fields such as tissue engineering, regenerative medicine, and in vitro cell culture models. These findings provide insights into cell‒matrix interactions and how these interactions regulate mechanically sensitive molecular pathways within cells.^36^ The composition of ECM can affect YAP1 activity. For example, matrix components such as collagen and elastin can promote the nuclear localization and activity of YAP1, while laminin can inhibit its nuclear localization and activity.^37^ Cells are sensitive to extracellular hydrostatic pressure and control the cellular volume and homeostasis in various diseases. YAP1/TAZ are cotranscription factors in Hippo-pathway^38^ and regulated by interstitial fluid pressure (IFP).^39^ This phenomenon is highly relevant in diseases characterized by elevated IFP, most notably solid tumors. Since high IFP blocks drug delivery and promotes progression, targeting YAP/TAZ could disrupt this mechanopathological loop. Strategies to inhibit YAP/TAZ may not only reduce tumor growth but also normalize IFP, thereby improving chemotherapy efficacy. YAP1 is activated in cardiomyocytes to promote the cell cycle upon hypertensive stress.^40^ This finding reveals YAP1 is a critical mediator linking mechanical stress to the heart's hyperplastic and hypertrophic responses. It highlights a tantalizing yet complex therapeutic opportunity: harnessing this pathway for cardiac regeneration while carefully avoiding its role in promoting pathological heart disease.

The upstream signals including cellular polarity, tissue stiffness, and mechanical forces, regulate the activity of YAP1/TAZ.^41^ Cell polarity^42^ is an important factor in regulating YAP1 activity. During the establishment of cell polarity, the expression of Crumbs complex and Scribble complex is regulated, thereby affecting YAP1 activity. The crumbs complex and Scribble complex form patches on the cell membrane that promote the phosphorylation and inhibition of YAP1 activity. The multi-input regulation of YAP1/TAZ underscores a paradigm shift in biology: cell fate is orchestrated by the seamless integration of physical and chemical signals. This reveals profound insights for regenerative medicine and oncology. In cells, mechanical stress plays a fundamental role in influencing the establishment and maintenance of cell polarity. For instance, biophysical forces can drive the organization of polar proteins into membraneless condensates with distinct physiological functions, a mechanism that contributes to symmetry breaking and polarity establishment even in bacterial cells. Moreover, cell polarity, often regarded as a “fate-determining” spatial cue, is itself modulated by mechanosensitive signaling molecules such as YAP1. These observations highlight a tightly interconnected regulatory network among mechanical stress, cell polarity, and YAP1 activity, which collectively orchestrate essential cellular processes, including growth, differentiation, and migration.

 Mechanical stress activates downstream molecules through multiple signaling pathways to drive disease pathogenesis. In prostate cancer, FSS is sensed by PIEZO1-Src-YAP1 axis to influence cell biological behaviors.^43^ The direct PIEZO1-Src-YAP1 axis suggests a rapid, potentially Hippo-independent pathway that may prioritize immediate proliferative or survival responses in a tumor setting. This finding aligns with the role of YAP1 as an oncogene, where bypassing canonical regulatory checkpoints could be advantageous for cancer progression. In periodontal ligament (PDL) cells, FSS activates P38, which promotes the phosphorylation of AKT to influence the nuclear transduction of YAP1.^44^ Conversely, the pathway involves a more complex cascade. The involvement of P38, a kinase often associated with stress response and differentiation, might indicate a more regulated signaling process. This could fine-tune YAP1 activity to maintain tissue homeostasis and promote appropriate adaptive remodeling in response to mechanical forces from chewing. In liver cancer, the mechanotransducer YAP1 induces target genes associated with epithelial‒mesenchymal transition (EMT) and enhances cancer metastasis.^45^ This finding positions YAP1 not only as a passive biomarker but also as an active effector that translates mechanical cues, such as increased liver stiffness from fibrosis to prometastatic transcriptional programs. This explains why hepatocellular carcinoma (HCC), which often arises in a stiff fibrotic microenvironment, is highly metastatic. The nuclear transduction of YAP1 is induced by ECM stiffness to increase anoikis resistance and cancer progression.^46^ The ECM stiffness activates the integrin–FAK–CDC42 signaling pathway to promote the nuclear transcription of YAP1 in bladder cancer (BC).^47^ Once YAP1 is activated in nuclear, its downstream target molecules are highly expressed, such as lysyl oxidase (LOX). LOX family members can increase the crosslinking of collagen in ECM and matrix stiffness.^48^ This evidence proves that tumors can mechanically manipulate their own microenvironment to create a progressively stiffening niche that fuels their own growth and survival. Breaking this YAP1-LOX-mechanical feedback loop represents a promising therapeutic strategy for cancers.

Taken together, this collective evidence underscores that mechanical signaling is not a peripheral pathway but a central driver of cell behavior. YAP1 emerges as the master integrator, and its pathological activation creates a stiffened, protumorigenic niche. Breaking the mechanical feedback loop offers a novel and compelling approach for cancer treatment.

#### Integrin signaling

Integrin-mediated sensing of biochemical and biophysical cues from the ECM serves as a primary regulator of YAP1/TAZ activity. We summarize the core molecular mechanisms through which integrin signaling activates YAP1/TAZ, highlighting key pathways involving FAK, SRC kinase, PI3K-AKT, Rho GTPases and mechanical force transduction. Understanding these mechanisms provides critical insights into tissue homeostasis, regeneration, and diseases such as cancer and fibrosis.^49^

Upon integrin clustering and activation of focal adhesions, FAK undergoes autophosphorylation at Tyr397, creating a binding site for SRC family kinases. The FAK–SRC complex then phosphorylates and activates PI3K, leading to the generation of phosphatidylinositol (3,4,5)-trisphosphate (PIP3) and subsequent activation of AKT. AKT directly phosphorylates and inhibits LATS1/2, the core kinase of the Hippo pathway, thereby preventing YAP1/TAZ phosphorylation and promoting their nuclear accumulation.^50^ The Integrin-FAK-YAP1 pathway is a typical route in cell progression that regulates cell behaviors such as proliferation, invasion, and migration.^51^ The ITGB1/FAK/YAP1^52^ complex mediates drug resistance at the surface of gastric cells through typical extracellular vesicles.^53^ Integrin engagement activates RhoA through guanine exchange factors (GEFs), such as GEF-H1. RhoA, through its effector ROCK, promotes actin polymerization and stress fiber formation. A robust F-actin network inhibits LATS kinase activity and sequesters inhibitory proteins of YAP1/TAZ, facilitating YAP1/TAZ nuclear translocation. This represents a Hippo-independent mechanism of YAP1/TAZ regulation.^54^ ITGβ3 triggers podocyte injury via RhoA–YAP1 apoptosis axis in atherosclerosis, and high-glucose treatment enhances this process.^55^ Integrins serve as mechanosensors that transmit mechanical forces from the ECM to the cytoskeleton. Under high mechanical tension, force-dependent reinforcement of integrin–FAK signaling and Rho activation further enhances YAP1/TAZ nuclear localization. On soft matrices, low cytoskeletal tension results in YAP1/TAZ cytoplasmic retention and inactivation. Mechanistically, the soft ECM inhibits the progression of cardiac reprogramming through the integrin, ROCK, and YAP1 signaling pathways. Therefore, mechanical transduction may offer potential therapeutic targets for treating cardiac diseases.^56^ While integrin signaling can bypass the canonical Hippo kinase cascade, it significantly cross-talks with the pathway by inhibiting LATS1/2. Phosphorylation of LATS by FAK-SRC and AKT represents a key integration node where adhesion-derived signals directly modulate Hippo pathway activity.

#### Cytoskeleton

The cytoskeleton is composed of proteins such as microtubules, actin, and intermediate filaments. These proteins can affect YAP1 activity by regulating its interaction with the cytoskeleton. In periodontal ligament stem cells (PDLSCs), lipoprotein receptor-related protein 6 (LRP6) converts mechanical signaling into molecular mechanism via the F-actin/YAP1 pathway to influence alveolar bone remodeling, as described in reference.^57^ This F-actin/YAP1 pathway activation ultimately regulates the expression of genes that control alveolar bone remodeling (bone resorption and formation). This is the fundamental process that allows teeth to move orthodontically and for the surrounding bone to be reshaped. Kank1 plays a crucial role in cell adhesion, actin polarization, and the activation of YAP1 during myoblast proliferation.^58^ Kank1 plays an essential role as a mechano-regulatory hub that synchronizes physical cues with biochemical signals to drive myoblast proliferation, a process vital for muscle biology. MiR-320-3p regulates myogenesis by targeting CFL2, a key factor required for F-actin polymerization and polarization. Consequently, the overexpression of miR-320-3p downregulates CFL2 expression, leading to disrupted actin cytoskeletal dynamics. This impairment in actin organization subsequently attenuates the nuclear translocation and transcriptional activity of YAP1, a mechanosensitive effector critical for myogenic progression.^59^ Microtubule disruption often promotes actin polymerization and RhoA activation, indirectly enhancing YAP1 activity. Dynamic interactions between actin, microtubules, and intermediate filaments ensure coordinated cellular responses to mechanical cues, which are integrated and amplified through YAP1-driven transcriptional programs. In melanoma, YAP1 promotes malignant biological behavior by regulating the target molecule actin-related protein 2/3 complex subunit 5 (ARPC5).^60^ Meanwhile, as a transcriptional coactivator, YAP1 directly binds to transcription factors such as TEAD to enhance the expression of cytoskeleton-related genes. This regulatory function establishes a critical feedback loop that reinforces cellular mechanical properties and promotes YAP1-driven biological processes.

#### Growth factors

Some growth factors, such as platelet-derived growth factor (PDGF) and fibroblast growth factor (FGF), can stimulate YAP1 activity. These growth factors can bind to their receptors and trigger a series of signaling pathways that ultimately lead to the phosphorylation and nuclear localization of YAP1.^61^ The activated receptor complexes initiate several key pathways that collaboratively regulate YAP1. The recruited adaptor proteins directly activate PI3K. PI3K generates the lipid second messenger PIP3, which recruits AKT to the plasma membrane. AKT can directly phosphorylate YAP1 on specific serine residues.^62^ The recruited complex activates the small GTPase Ras. ERK can phosphorylate YAP1, influencing its stability and cotranscriptional activity. The effects are complex and can be context-dependent, but often contribute to YAP1's pro-growth functions. Activated RhoA stimulates actin polymerization and the formation of stress fibers.^63^^,^^64^ A stiff, polymerized actin cytoskeleton is one of the most powerful signals for YAP1 activation. This mechanism elegantly demonstrates how extracellular growth signals are transduced into intracellular mechanical and biochemical changes that directly control the nuclear transcriptional machinery, with YAP1 acting as a central hub.

#### G protein-coupled receptors (GPCRs)

GPCRs can bind to a variety of ligands, including lipids, peptides, and amino acids. The binding of ligands to GPCRs can activate multiple signaling pathways, including the MAPK and PI3K, which can affect YAP1 activity.^65^ GPCRs do not typically phosphorylate YAP1 directly. Instead, they modulate the activity of the core kinase cascade of the Hippo pathway, which is the primary kinase responsible for inactivating YAP1 by phosphorylating it on serine 127, leading to its cytoplasmic sequestration.^66^ This mechanism is fundamental to processes ranging from organ development and tissue regeneration to cancer progression, where aberrant GPCR signaling often leads to sustained YAP1 activation.

### Nuclear mechanism: transcriptional coactivation

Because of lacking DNA binding regions, the transcriptional enhancer factors TEF 1−4 are activated by YAP/TAZ to increase gene expression.^67^ TEAD transcription factors can bind to YAP1 and promote its transcriptional activity.^68^ Numerous genes associated with malignant biological behaviors are regulated by YAP/TAZ-TEAD binding.^69^ The transcription factors SOX-5 and SOX2 can interact with YAP1 to sustain proliferation signaling pathways in NSCLC cells.^70^ The expression of autophagy-related gene P62 is regulated by YAP1, which is mediated by the ERK signaling pathway.^71^ The cell cycle-related genes CLK and PLK are more downregulated by YAP1 knockdown.^72^ There was a significant positive correlation between YAP1 expression and interstitial-related markers, such as vimentin, *N*-cadherin, SNAI1, and SNAI2.^73^ In the cancer process, YAP1 is involved in tumor immunity and regulates the expression level of PD-L1.^74^ This passage depicts YAP1 as a master regulator. Positioned downstream of multiple signaling pathways (such as Hippo and ERK) and upstream of gene transcription, it orchestrates the malignant progression of cancer in an all-encompassing manner by regulating the expression of a series of critical genes involved in processes ranging from cell proliferation and metabolism to migration and immune modulation. This makes it an extremely attractive potential therapeutic target in cancer treatment.

In summary, YAP1 and TAZ are not mere passive effectors but dynamic and sophisticated integrators of cellular signaling. Their activity is exquisitely controlled by a dual-layered regulatory system: a core phosphorylatory switch governed by the Hippo pathway and a broader layer of regulation involving mechanotransduction, cellular metabolism, and GPCR signaling. In the nucleus, their partnership with TEADs and other factors, coupled with their ability to recruit powerful chromatin-modifying machinery, allows them to execute a precise transcriptional program that dictates cell fate. Understanding these intricate mechanisms is paramount for developing therapeutic strategies targeting YAP1/TAZ in human diseases, particularly cancer.

## The multifaceted role of YAP1 as a master regulator: bridging autophagy, ferroptosis, metabolism, and tumor immunity

### Crosstalk with YAP1 and autophagy

YAP1, a pivotal transcriptional coactivator, is integrated into diverse biological processes, including proliferation, apoptosis, invasion, migration, autophagy, and metabolism. However, its role in autophagy appears context-dependent and warrants critical examination. For instance, in HCC, YAP1-mediated inhibition of autophagy synergizes with anti-PD-L1 therapy, suggesting a potential immunomodulatory mechanism that may enhance therapeutic efficacy.^75^ During the formation of autophagosomes, YAP1 and nuclear factors play significant roles.^76^ Conversely, under bacterial infection conditions, YAP1/TAZ promotes autophagy to counteract *Staphylococcus aureus*, highlighting its role in cellular defense.^77^ This functional duality raises important questions about the tissue- and stimulus-specific regulation of autophagy by YAP1. Further complexity is observed in cancer models. FSS activates the RhoA/YAP1 axis to induce promigratory autophagy in cancer cells.^78^ Whereas in colorectal cancer, YAP1/TEAD suppresses autophagy by upregulating the antiapoptotic protein Bcl-2.^79^ These seemingly contradictory findings underscore the necessity to delineate the precise molecular determinants, such as upstream signals, binding partners, and epigenetic states, that dictate YAP1's functional output in autophagy.

Beyond mere phenomenological observation, key questions remain unresolved: How does YAP1 reconcile dual roles in both promoting and inhibiting autophagy? What is the tissue-specific effector that dictates this functional switch? Addressing these conflicts is not only essential for mechanistic understanding but also for developing context-aware therapeutic strategies targeting the YAP1–autophagy axis in cancer and infection.

### Crosstalk with YAP1 and ferroptosis

The regulation of ferroptosis by YAP1 exhibits considerable context-dependent complexity, particularly across different pathological conditions. In non-small cell lung cancer (NSCLC), HIF-1α-mediated suppression of the Hippo pathway leads to YAP1 activation and subsequent ferroptosis inhibition, suggesting a potential mechanism for tumor cell survival under hypoxic conditions.^80^ Conversely, in sepsis-associated acute lung injury, YAP1 also acts as a negative regulator of ferroptosis, indicating a protective role in inflammatory tissue damage.^81^ This functional duality raises important questions about the tissue- and disease-specific roles of YAP1 in regulating cell death processes. Further adding to this complexity, YAP1 can also promote ferroptosis sensitivity through its transcriptional target SKP2, an E3 ubiquitin ligase.^82^ This apparent contradiction highlights the critical need to identify the precise molecular determinants that dictate YAP1's function as either a suppressor or promoter of ferroptosis. The p53-YAP1 axis has emerged as a key signaling node in this regulatory network, as demonstrated in colon cancer, where cytoglobin-induced ferroptosis depends on YAP1 knockdown.^83^ While intercellular communication mediated by the NF2/YAP1 pathway further fine-tunes this process.^84^ These seemingly conflicting findings underscore the importance of microenvironmental cues, the cellular context and upstream signaling networks in shaping YAP1's functional outcomes. Future research should prioritize elucidating the specific conditions under which YAP1 switches its role in ferroptosis regulation, as this understanding will be essential for developing targeted therapeutic strategies that manipulate YAP1-mediated ferroptosis in cancer, inflammatory diseases, and beyond.

### Crosstalk with YAP1 and metabolism

YAP1 exhibits pleiotropic roles across physiological and pathological processes, though its functional consequences appear highly context-dependent. In metabolic disorders such as fatty liver disease, YAP1 activation mitigates lipid droplet accumulation and disease progression, suggesting a protective regulatory function.^85^ Conversely, in fungal models like *Aspergillus terreus*, YAP1 modulates reactive oxygen species mechanisms, highlighting its evolutionary conserved role in the stress response.^86^ This functional versatility extends to development and disease: YAP1 contributes to embryonic stem cell differentiation through NEDD4 mediation,^87^ while in vascular pathologies, YAP1/TAZ signaling is implicated in inflammatory activation, phenotypic switching, and aneurysm formation.^88^ Notably, its anti-inflammatory activity in certain contexts further underscores its therapeutic potential.^89^ These observations collectively point to a critical, yet unresolved question: what mechanisms underlie the stark functional divergence of YAP1 in different biological settings? The dual role of YAP1, which acts as either a disease-promoting or protective factor, may be influenced by cell type-specific binding partners, posttranslational modifications, or tissue microenvironmental cues. Future studies should prioritize elucidating these determinants, particularly through comparative analyses across models and conditions. Such insights will not only resolve apparent contradictions in the literature but also inform the development of context-aware therapeutic strategies targeting the YAP1 signaling network.

### The relationships between YAP1 and immune infiltration

The interplay between YAP1 and tumor immunology reveals complex, and at times contrasting, mechanisms that may either support or inhibit antitumor immunity, presenting both challenges and opportunities for therapeutic intervention. While ionizing radiation (IR) and YAP1 inhibition synergize to promote immunogenic cell death (ICD), enhance CD8⁺ T cell infiltration, and improve survival in patients with pancreatic cancer.^90^ YAP1 also correlates positively with favorable immune biomarkers, such as tumor mutation burden (TMB) and infiltration of CD8⁺ T, NK, and Th1 cells.^91^ This apparent duality underscores a context-dependent role for YAP1 in immune regulation. Further complicating this picture, YAP1 activation has been implicated in immune resistance mechanisms. In solid tumors, nuclear YAP1 driven by IFN-*γ* promotes phase separation and contributes to immunotherapy resistance.^92^ Similarly, YAP1 upregulates PD-L1 transcription in EGFR-TKI-resistant lung adenocarcinoma (LUAD) cells,^93^ yet in pancreatic cancer, DCLK1 suppresses PD-L1 via the Hippo pathway, highlighting tissue-specific regulatory circuits.^94^ Moreover, YAP1 modulates immunosuppressive cell populations: it is involved in Treg-mediated immune homeostasis^95^ and facilitates the recruitment of myeloid-derived suppressor cells (MDSCs) to support tumor progression.^96^ These findings collectively emphasize the dual nature of YAP1 as both an immune facilitator and an immune suppressor. Key questions remain unresolved: What determinants dictate whether YAP1 promotes T-cell infiltration or recruits immunosuppressive cells? Can we therapeutically decouple its protumorigenic functions from its potential immunostimulatory roles? Addressing these questions will be essential for developing novel combination therapies that leverage YAP1 targeting to overcome immune resistance and enhance the efficacy of existing immunotherapies.

## Epigenetic regulation of YAP1: implications for cancer progression and therapeutic opportunities

YAP1 has emerged as a central node intricately regulated by diverse epigenetic and mechano-biological mechanisms across pathological contexts, though significant questions remain regarding the integration and therapeutic targeting of these complex interactions. In spinal cord injury, METTL3-mediated m6A modification stabilizes YAP1 transcripts to promote cell survival,^97^ while in inflammation, melatonin suppresses DNMT1 to elevate miR-16b, thereby inhibiting YAP1 and attenuating inflammatory responses.^98^ Moreover, a recent study revealed that the Hippo/YAP1–TET1 axis mediates sorafenib resistance in HCC by epigenetically regulating the DNA repair gene program.^99^ This finding further underscores the potential of targeting YAP1 and its associated epigenetic regulatory network to overcome therapeutic resistance. Beyond RNA and DNA methylation, histone modifications also contribute to YAP1-driven oncogenesis. For instance, SETD1A, a histone H3K4 methyltransferase, has been shown to transcriptionally activate YAP1, thereby promoting primary resistance to sorafenib in hepatocellular carcinoma.^100^ This finding expands the repertoire of epigenetic regulators that converge on YAP1 to mediate therapy resistance. Notably, the regulatory relationship between YAP1 and the epigenetic machinery is bidirectional. While epigenetic modifiers regulate YAP1's activity, YAP1 itself can orchestrate large-scale epigenetic remodeling to enforce its oncogenic transcriptional program. A seminal study by Wu et al demonstrated that YAP1 directly promotes the expression of the DNA demethylase TET1. This YAP1–TET1 axis induces widespread DNA hypomethylation and chromatin accessibility changes, which in turn activate a protumorigenic gene expression signature that drives liver overgrowth and HCC development.^101^ This establishes YAP1 not only as a product of epigenetic regulation but also as a master regulator of the epigenetic landscape, creating a powerful feed-forward loop that amplifies its oncogenic signal. The capacity of YAP1 to function as a master epigenetic regulator is further highlighted by its direct role in remodeling chromatin architecture. Fetiva et al demonstrated that oncogenic YAP1 binding directly induces increased chromatin accessibility and activates enhancer activity at key genomic loci. This widespread reprogramming of the epigenome drives the expression of a potent transcriptional program enriched in cell cycle and cell migration genes, thereby fundamentally promoting malignant phenotypes.^102^ This mechanism, alongside the YAP1–TET1 axis, underscores how YAP1 dictates cell fate by orchestrating the epigenetic landscape. The interplay between YAP1 and the epigenetic machinery can also manifest as a direct partnership to repress tumor-suppressive programs. A compelling example is provided by Xu et al., who demonstrated that in gallbladder cancer, the YAP1/TAZ complex physically interacts with DNMT3A, a de novo DNA methyltransferase. This cooperative complex is recruited to the promoters of specific metastasis suppressor genes, where it catalyzes hypermethylation and transcriptional silencing, thereby powerfully driving cancer metastasis.^26^ This mechanism reveals that YAP1/TAZ can function as a scaffold, recruiting epigenetic writers to precisely silence gene expression, which adds another sophisticated layer to the epigenetic regulation of YAP-driven oncogenesis.

 Mitochondrial stress further engages YAP1-associated enhancers to drive epigenetic adaptation,^103^ highlighting the role of YAP1 as a multifunctional effector in stress and disease. Notably, mechanical cues and epigenetic modifications converge extensively in YAP1 regulation. Matrix stiffness activates integrin-mediated mechanotransduction, cytoskeletal remodeling, and nuclear signaling to promote YAP1 activation,^104^ while also inducing epigenetic alterations such as DNA methylation in gastric cancer.^19^ These processes are not isolated; mechanical signals can modulate miRNA expression in osteoarthritis^105^ and influence epigenetic states in arterial stiffness,^106^ suggesting broad crosstalk between biophysical and biochemical YAP1 regulatory mechanisms. Nevertheless, it remains unclear how mechanical inputs are precisely translated into context-specific epigenetic changes, and whether this crosstalk represents a targetable axis in fibrotic, malignant, or degenerative diseases. In cancer, post-translational regulation of YAP1 adds further complexity. USP47 enhances YAP1 stability in colorectal cancer and correlates with poor prognosis,^107^ whereas circRNAs and m6A modifications, particularly those mediated by METTL3—orchestrate YAP1 expression in hepatocellular carcinoma to drive vasculogenic mimicry and drug resistance.^108^^,^^109^ Similar METTL3-driven mechanisms promote YAP1 translation and chemoresistance in NSCLC^110^^,^^111^ and contribute to lenvatinib resistance via FZD10/β-catenin/YAP1 signaling.^112^ The inflammatory microenvironment further reshaped the m6A epitranscriptome of YAP1, enhancing its oncogenic function in LUAD.^113^^,^^114^ Paradoxically, METTL14 appears to suppress YAP1 in kidney injury,^115^ highlighting enzyme- and tissue-specific roles that remain poorly reconciled. These findings collectively position YAP1 at the intersection of mechanobiology, epitranscriptomics, and disease pathogenesis. Key challenges include resolving the contextual roles of m6A writers such as METTL3 versus METTL14, determining how mechanical signals are epigenetically encoded, and determining whether targeting YAP1-regulatory enzymes or mechanical cues offers viable strategies for modulating YAP1 in disease. Future studies should prioritize integrated multiomics and mechanobiology approaches to translate these mechanistic insights into targeted epigenetic or biomechanical therapies.

## Targeting the epigenetic‒mechanical axis: a novel therapeutic strategy in oncology

Cancer progression is orchestrated not only by genetic mutations but also through dynamic crosstalk between epigenetic remodeling and mechanical regulation. Epigenetic mechanisms, such as DNA methylation, histone modifications, and noncoding RNA activity, modulate gene expression and cellular phenotypes without altering DNA sequences, frequently contributing to malignant transformation, tumor heterogeneity, and therapy resistance. Simultaneously, mechanical regulation, which is mediated by ECM stiffness, FSS, and cytoskeletal tension, directly influences oncogenic pathways by modulating cell growth, differentiation, and metastatic dissemination. Emerging research reveals a tightly integrated bidirectional interplay between these two domains. Epigenetic alterations can dictate cellular mechanosensitivity; for example, DNA methylation patterns regulate the expression of mechanosensory genes (integrins, YAP1/TAZ), thereby shaping how cells perceive and adapt to mechanical cues. Conversely, mechanical stimuli can induce epigenetic reprogramming: nuclear deformation resulting from elevated ECM stiffness modulates nuclear pore permeability, histone modifications, and DNA methylation, ultimately affecting transcriptional outputs and promoting malignant phenotypes.

In cancer, this mechano-epigenetic dialogue drives key pathological processes. Metabolic heterogeneity and DNA damage response (DDR) activation are closely linked to combined mechanical and epigenetic dysregulation. Moreover, the mechanical properties of tumors, such as increased stiffness, not only arise from malignancy but also actively sustain it by reinforcing protumor epigenetic states. Targeting this synergistic axis offers transformative therapeutic potential. Strategies may include: using epigenetic drugs to disrupt mechanical memory and sensitize tumors to biomechanical cues; Modulating tumor mechanics via stromal targeting or mechanotherapy to reverse oncogenic epigenetic programming; Developing combination therapies that concurrently inhibit mechanosignaling and epigenetic modifiers to suppress metastasis and overcome resistance. Advancing these approaches will require innovative biomimetic models and mechano-epigenetic biomarkers to decode context-specific interactions. Ultimately, integrating epigenetic and mechanical targeting may unlock novel strategies for precision oncology, disrupting the physical and molecular networks that underlie cancer progression.

## Conclusion and future perspectives

In this review, we systematically examined the multifaceted roles of YAP1/TAZ in cancer, with a particular emphasis on their interplay with key biological processes, including autophagy, ferroptosis, metabolism and immune infiltration. As central effectors of the Hippo pathway, YAP1/TAZ are critically involved in the.^116^ Their activity is robustly modulated by diverse mechanical cues, such as ECM stiffness, FSS, and cytoskeletal tension, which promote the nuclear translocation of YAP1/TAZ and the activation of downstream signaling cascades, including Wnt/β-catenin, Notch, and TGF-*β*.^117^ These mechanical inputs are transduced into biochemical signals that influence cell morphology, polarity, and phenotypic behavior,^118^ ultimately driving cancer hallmarks such as proliferation, invasion, metastasis, and resistance to therapies, including EGFR-TKIs.^119^ In particular, ECM stiffness, a widely studied mechanical factor in pathological contexts,^120^^,^^121^ activates integrin-mediated signaling and enhances YAP1/TAZ nuclear accumulation.^122^ Recent advances further underscore that mechanical cues can induce epigenetic modifications that regulate YAP1/TAZ activity, unveiling a novel dimension of mechano-epigenetic crosstalk in cancer progression.^123^

Beyond traditional signaling, YAP1/TAZ are implicated in emerging cancer-related processes such as autophagy, ferroptosis, cuproptosis, mitochondrial metabolism, and oxidative stress,^124^ highlighting their broad regulatory scope. Additionally, noncoding RNAs, which function as upstream regulators or downstream effectors of YAP1/TAZ, offer promising therapeutic avenues for perturbing YAP1/TAZ signaling networks.^125^ Targeting these RNAs may overcome the limitations of conventional treatments and improve patient outcomes.

A deeper understanding of the molecular mechanisms linking mechanotransduction and epigenetic regulation of YAP1/TAZ will accelerate the development of novel cancer therapeutics. Future efforts should focus on elucidating mechanosensitive molecules and their epigenetic modifiers within the YAP1/TAZ signaling network and validating their functional roles using physiologically relevant animal models. We hope this review stimulates further exploration of YAP1/TAZ mechano-epigenetic cross-talk and promotes the translation of mechanobiology-informed strategies into clinical applications for cancer treatment.

**Abbreviations**
TME: tumor microenvironment
ECM: extracellular matrix
FAKs: focal adhesion kinases
YAP1: Yes-associated protein 1
m6A: N6-methyladenosine
WSS: wall shear stress
FSS: fluid shear stress
IFP: Interstitial fluid pressure
PDL: periodontal ligament
EMT: epithelial-mesenchymal transition
HCC: hepatocellular carcinoma
BC: bladder cancer
LOX: lysyloxidase
PIP3: phosphatidylinositol (3,4,5)-trisphosphate
GEFs: guanine exchange factors
PDLSCs: periodontal ligament stem cells
LRP6: lipoprotein receptor-related protein 6
ARPC5: actin-related protein 2/3 complex subunit 5
PDGF: platelet derived growth factor
FGF: fibroblast growth factor
GPCRs: G protein-coupled receptors
NSCLC: Non-small cell lung cancer
IR: ionizing radiation
ICD: immunogenic cell death
TMB: tumor mutation burden
LUAD: lung adenocarcinoma
MDSCs: myeloid-derived suppressor cells
DDR: DNA damage response.

## Data Availability

All data discussed in this review were derived from previously published studies and publicly available datasets. Relevant sources were cited in the references.
